# A geo-computational algorithm for exploring the structure of diffusion progression in time and space

**DOI:** 10.1038/s41598-017-12852-z

**Published:** 2017-10-03

**Authors:** Wei-Chien-Benny Chin, Tzai-Hung Wen, Clive E. Sabel, I-Hsiang Wang

**Affiliations:** 1 Department of Geography, National Taiwan University, Taipei City, 10617 Taiwan; 2 Department of Environmental Science, Aarhus University, 4000 Roskilde, Denmark

## Abstract

A diffusion process can be considered as the movement of linked events through space and time. Therefore, space-time locations of events are key to identify any diffusion process. However, previous clustering analysis methods have focused only on space-time proximity characteristics, neglecting the temporal lag of the movement of events. We argue that the temporal lag between events is a key to understand the process of diffusion movement. Using the temporal lag could help to clarify the types of close relationships. This study aims to develop a data exploration algorithm, namely the TrAcking Progression In Time And Space (TaPiTaS) algorithm, for understanding diffusion processes. Based on the spatial distance and temporal interval between cases, TaPiTaS detects sub-clusters, a group of events that have high probability of having common sources, identifies progression links, the relationships between sub-clusters, and tracks progression chains, the connected components of sub-clusters. Dengue Fever cases data was used as an illustrative case study. The location and temporal range of sub-clusters are presented, along with the progression links. TaPiTaS algorithm contributes a more detailed and in-depth understanding of the development of progression chains, namely the geographic diffusion process.

## Introduction

A geographic diffusion process is the evolution of space-time clusters of entities. Geographic diffusion processes are a scientific field of research that focuses on the movement of events, goods, information, ideas, or people through space and time^1,2^, that is, how do things spread from one place to another through time. In the literature, there are three types of diffusion: contagious, relocation, and hierarchical^3–5^. Contagious diffusion is concerned with proximate contact and is highly influenced by the friction of distance. Relocation processes involve larger leaps in spatial distance. Hierarchical diffusion is influenced by inherent hierarchies of geographical space, such as demographic, socio-economic, or the mobility structure of a region. While modeling geographic diffusion processes from the original event point locations, there are two critical points to consider: the occurrence of events, and the transmission of events.

The concept of event occurrence focuses solely on the spatial-temporal locations of events. To model the process of diffusion, first, we need to know where and when events occurred. For example, the onset date and the residential or working locations of the patients of a disease outbreak have to be recorded and analyzed in the model. Point pattern analysis methods are designed to describe the pattern of the locations of the events, such as disease cases^6–8^, accidents^9^, crime locations^10,11^, and disaster locations^12,13^. Point pattern analysis can be classified into distance-based or density-based techniques^1^. Distance-based techniques, such as nearest neighbor analysis, use information on the spacing of points to define a pattern^14,15^. Density-based techniques, such as quadrat analysis and kernel density estimation, rely on various characteristics of the frequency distribution of the observed numbers of points in regularly defined sub-regions in the study area^16,17^. In spatial epidemiology, kernel density estimation has been used to estimate the spatial distribution of potential risk factors^7^. For example, Sabel *et al*.^7^ mapped the spatial distribution of the relative risk based on patients’ residential locations, and spatial temporal trends of the groups of patients based on their age groups. In summary, point pattern analysis detects spatial clustering^18–22^ and describes the spatial pattern of the occurrences of events.

On the other hand, the concept of the transmission of events focuses on movement through space and time. Diffusion of diseases has been studied for decades. Using Iceland as the study area, Cliff, Haggett and their team intensively worked in the 1960s on the spread of infectious disease within a closed island community in time and space^3,23–25^. They attempted to link epidemic models with spatial theory and had some success in revealing underlying mechanisms of movement of disease through time and space. Aside from modeling diffusion from space-time characteristics, recent studies have used graph theory and complex network analysis to explicitly model relationships between the events^26–28^. Transmission relationships were modeled at various scales of networks, which included individual social networks^29–31^, meta-population and sub-population networks^32,33^, buildings network^34^, and cities or countries networks^35,36^, by converting the objects of study into nodes and the contacts or interactions between them into links. Using complex network theories to analyze the transmission relationships provides clear topological structure of contacts in terms of nodes and links for revealing the process of complex interactions^37,38^. These studies attempted to understand the process of the exposure to disease through an agent- or equation-based simulation, or an integrated modeling approach. For example, Meloni *et al*.^32^ investigated infectious disease spread using a meta-population system, a network composed of subpopulations. They modeled changes in human movement behavior in response to the status of disease at the location, and simulated the transmission of disease under these scenarios. They presented the concept of an invasion tree that shows disease progression by defining a directional link from the origin to the destination subpopulations of an infection process. Disease diffusion in space and time has also been modelled by spatial dynamic models to understand the spatial pattern formation^39–41^. Spatial dynamic modeling is a mathematical approach that captures dynamic behaviors with patch-based spatial interaction models (i.e. cellular automata), for revealing population dynamic processes, such as the disease spreading, predator-prey interaction, and the interaction between population density and the fitness of individuals^42–44^. Therefore, we see that the major function of modeling the transmission between events is to understand and detect the movement process.

The temporal dimension is fundamental to understanding human activity^45^. Taking the temporal dimension into consideration is crucial for investigating space-time clustering and diffusion processes^46^. Previous studies of space-time point data analyses aim to investigate two spatial temporal phenomena: space-time interactions and space-time clustering. Space-time interactions determine whether a significant association between short distances in time and space exist. For example, the Knox test, a method which uses a critical space and time to determine whether a pair of events is spatially and temporally close. If the distances in space and time are correlated, a space-time interaction exists^47^. In the spatial epidemiological field, these tests can determine whether epidemics have contagious characteristics^48–50^. On the other hand, space-time clustering focuses on detecting clusters of events that are close with each other in both spatial and temporal dimensions. Space-time clustering methods can be used to detect a statistically significant excess of events occurring within a limited space-time continuum, which indicate where and when a situation becomes more serious. SaTScan, a space-time scan statistic method, which differs from space-time interaction tests, can identify when and where clusters are, and has been used to detect space-time clusters^51^. By considering the temporal dimension, not only the spatial location of the clusters but also the temporal periods of the clusters can be revealed.

Diffusion processes emphasize the movement of events through space and time^29^. But, neither space-time interactions nor space-time clustering phenomena are designed to capture the temporal differences of movements. Two events are considered related in space-time dimensions if they happen at the same place in the same time, i.e., two events happened in a small spatial range and temporal differences. However, while diffusion processes describe the spread of events through space and time, it means a temporal lag must be in between the source and target events, i.e., the second event should have occurred some time after the first event, and also not too far from it. This is the case especially in disease diffusion processes, where transmissions may experience a temporal lag for an incubation period, that is the time between infection and disease emergence. Thus a temporal lag between the transmission pairs should be considered in the understanding of disease diffusion^52^. To study disease diffusion processes, previous studies that used simulation approaches, including equation- and agent-based modeling, considered the shifting in temporal dimension as a key aspect in simulation models^53–56^. In disease diffusion simulation models, such as the susceptible-exposed-infectious-recovered (SEIR) model, a patient is exposed after physical contact with another infectious patient, and then waits for several time steps (depending on the particular disease etiology) before becoming an infectious patient^53^. The temporal lag effect has been considered in previous simulated diffusion studies, but the purpose of these studies was to understand the outcomes of different policy scenarios. In other words, a simulation approach cannot be used for empirical data exploration purpose.

From a data exploration perspective, the purpose of which is to identify patterns within space-time data, considering temporal lag can help clarify the relationships of space-time proximate events, and capture the progression of diffusion events. This study aims to develop a novel algorithm that utilizes temporal lag properties for understanding diffusion processes. Previous studies in diffusion have tended to focus on visualizing the spreading processes, whereas here we propose to extend this work by modeling the process, specifically to identify the space-time pattern and understand the structure and relationships between the clusters of events. First we describe the proposed algorithm, before we demonstrate its applicability using Dengue Fever data from Kaohsiung City, Taiwan.

## Method

**Figure 1:**
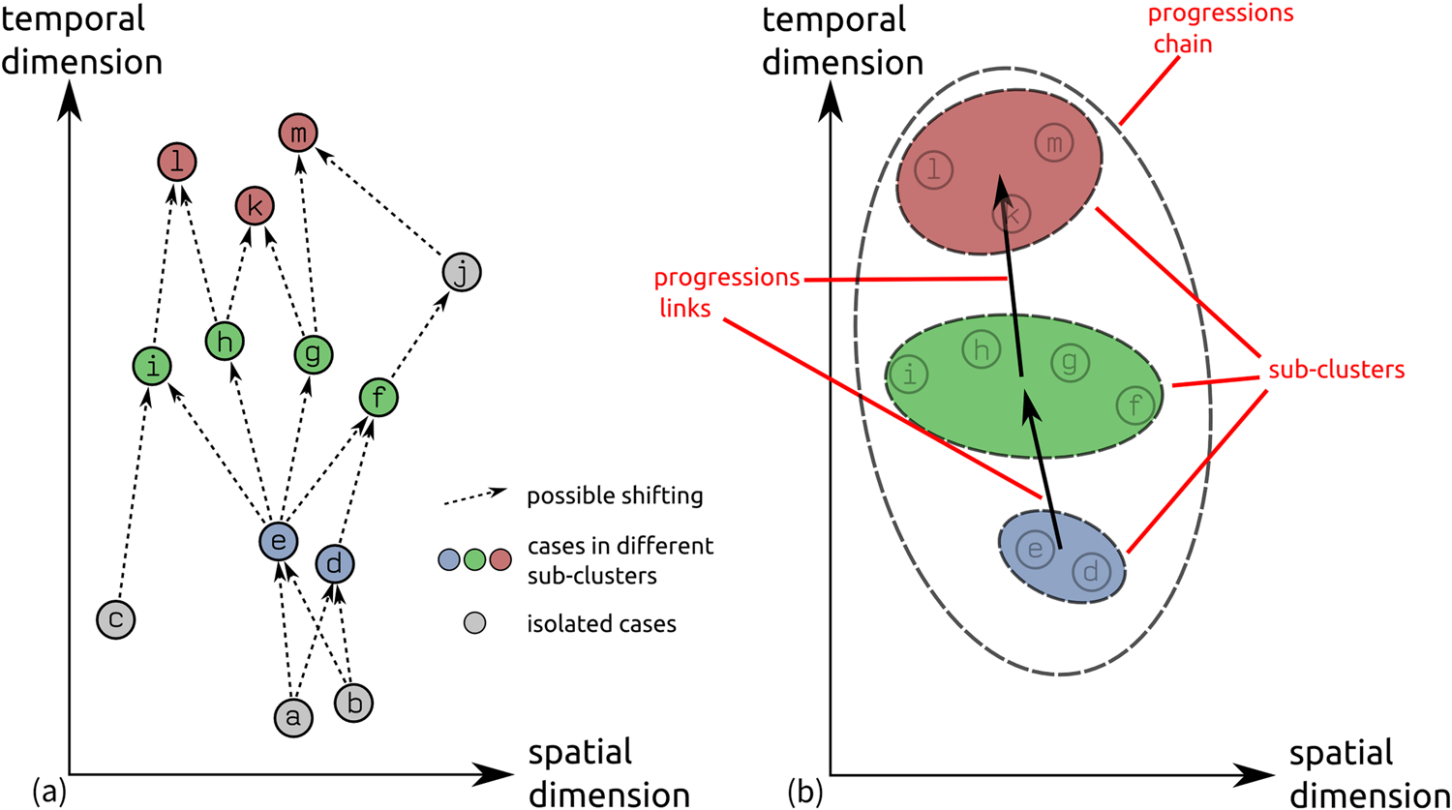
The illustration of sub-clusters in simplified space-time dimensions. In the figure, the cases all occur in a line on the spatial dimension, therefore the X-axis distance equals their spatial distance. (**a**) Illustrates the point distribution through space and time dimensions; (**b**) shows the sub-clusters, progression links, and the progression chain that are detected from the events distribution.

In this section, we present a novel algorithm, namely the TrAcking Progression in Time and Space (TaPiTaS) algorithm. We decomposed the diffusion process into sub-clusters and progression links between sub-clusters. The TaPiTaS algorithm uses the spatial and temporal distance between each pair of points to identify the most probable common origin and to detect sub-clusters. A sub-cluster is formed by a group of spatially and temporally close points that are probably related to one or several common origins. By common origin, we mean the source or in an epidemiological context, the original infective agent or individual that is common to all subsequent sub-clusters. Then, sub-clusters are connected by progression links according to the spatial and temporal relationships of these sub-clusters. Finally, progression chains that are formed by several linked sub-clusters could be revealed. The illustration of the sub-clusters is shown in Fig. 1, where the spatial dimensions are reduced to one dimension to show if the cases are close to each other.

**Figure 2:**
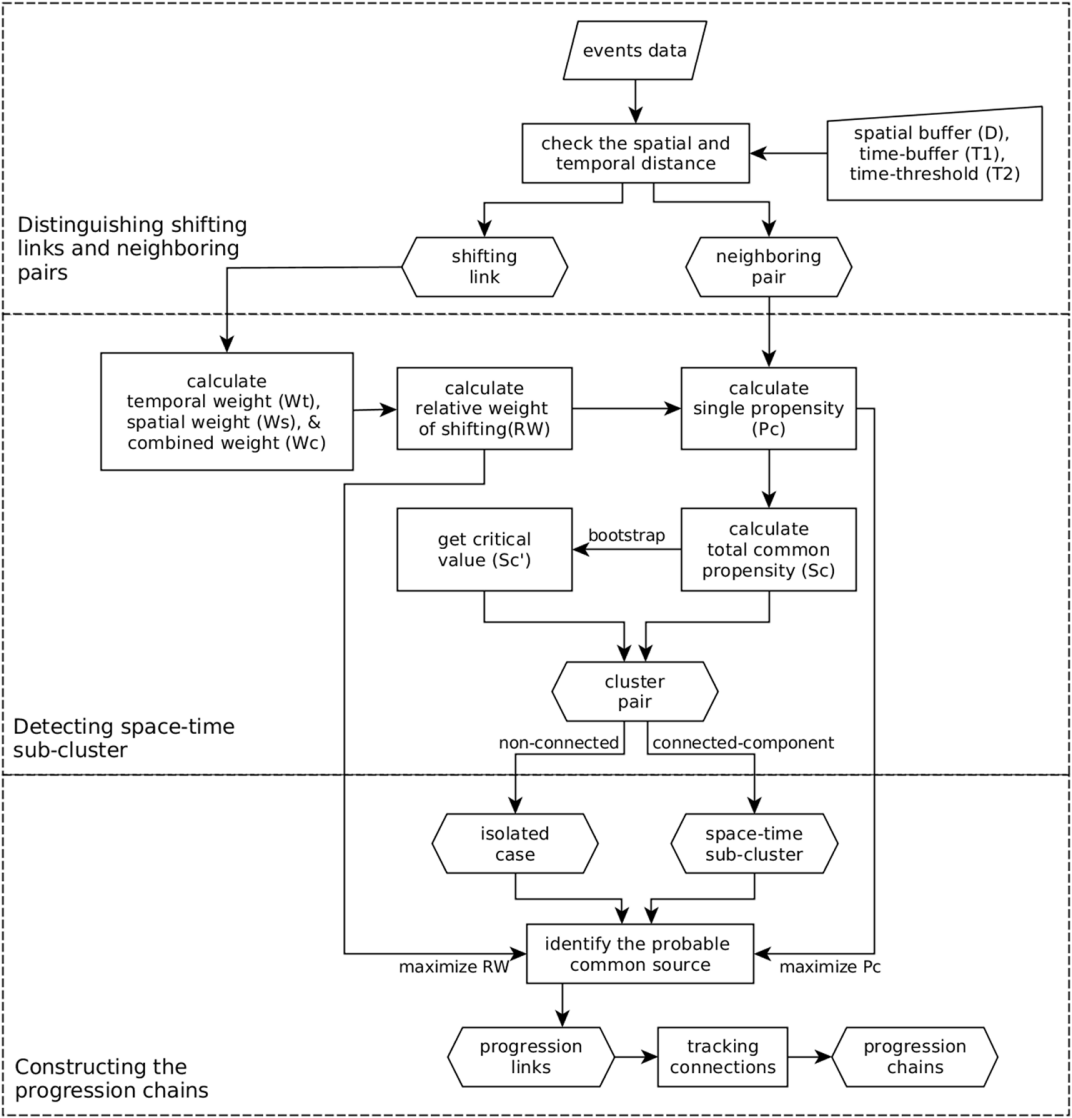
The calculation framework of the algorithm.

The TaPiTaS algorithm is composed of three steps. The first step distinguishes the relationships of each pair of the spatially close events into two types: shifting link or neighboring pair. The second step focuses on identifying space-time sub-clusters. The third step aims to construct the progressions between sub-clusters. The algorithm framework is shown in Fig. 2. We applied the TaPiTaS algorithm to individual cases of Dengue Fever from 1998 to 2015 in Taiwan.

### Distinguishing shifting links and neighboring pairs

While the spatial diffusion process is a concept describing the movements of events, it takes time to shift from one location to another. If two events happened in a same area at the same time, they can be considered as a space-time cluster of events, but not a spreading process. In point data analysis, the idea of the same area is captured by a spatial buffer zone, i.e., if one event happened within a distance buffer of another event, the two events are considered as spatially neighbors. For the temporal dimension, the idea of the same time can also be captured by a time buffer, i.e., if one event happened within a time buffer of another event, they are temporal neighbor. If two events happened in the same area, and the second event happened immediately after the time buffer from the first event, the second event can be considered as the outcome of the first event, that the first event has shifted to the location of the second event. This situation captures the concept of temporal lag, which is defined as the temporal interval that is needed for the outcome event to appear starting from the occurrence of the source event. But, if the second event happened a long time after the first event, they may be indirectly connected, but the relationship between the two events is not specified, thus, can be considered as not related.

**Figure 3:**
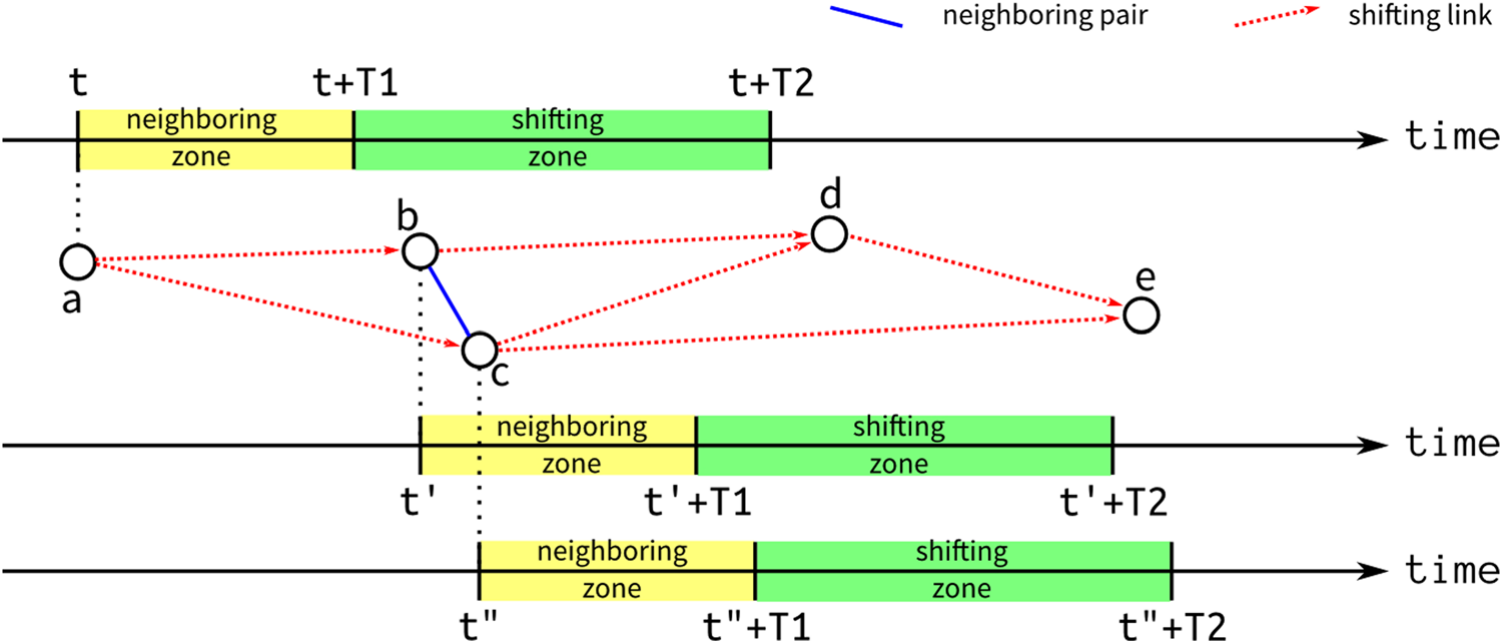
The searching time-buffer (*T*1) and time-threshold (*T*2) from the cases. The onset time of illness of cases a, b, and c are *t*, *t*′, and *t*′′, respectively. Both onset time of case b and c fall in the shifting zone of the case a (the range between *t* + *T*1 and *t* + *T*2), thus two shifting links (a,b) and (a–c) are identified. The shifting links (b–d), (c–d), (c–e), and (d–e) are found using the same concept. The onset time of case c falls inside the neighboring zone of case b (the range between *t*′ and $t^{\prime} +T1$), therefore the relationship between (b,c) is identified as a neighboring pair. There is no direct relationship between (a–d), (a–e), and (b–e), because the latter falls beyond the shifting zone of the former.

The first step of the algorithm is to separate the spatially neighboring events into the three types of relationships (Fig. 3). To those pairs of events happened within a spatial buffer (*D*), we denote the pair as a *neighboring pair* if the temporal-length (temporal lag) between the two events is shorter than or equal to a time-buffer (*T*1). We denote the pair as a *shifting link* if the time-lag between the two events is longer than the time-buffer but shorter or equal to a time-threshold (*T*2). And we denote the pairs with longer time-lag than the time-threshold as non-related, which are not included in the next procedure.

### Detecting space-time sub-clusters

**Figure 4:**
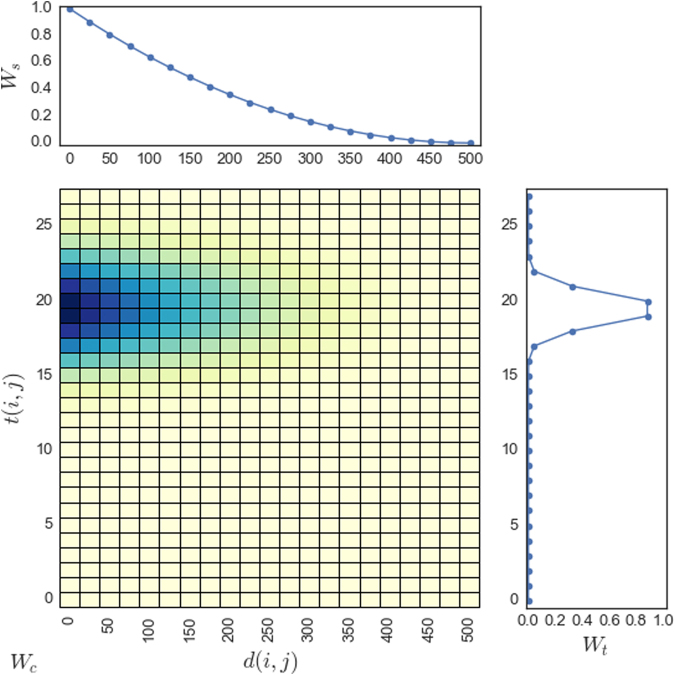
The illustration of the spatial (*W*_*s*_), temporal (*W*_*t*_), and combined (*W*_*c*_) weighting schemes. The top figure shows the spatial weight decrease with the increasing distance (calculated using Equation 2, *D* is set to 500); the figure on the right shows the temporal weight having a bell curve with the center around 19–20 (calculated using Equation 3, parameters are set to *T*1 = 12, *T*2 = 27); the bottom left figure shows the combined weight distribution using the colors, the darker the higher *W*_*c*_ (calculated using Equation 1).

To detect space-time sub-clusters, the algorithm analyzed neighboring pairs and shifting links. Then, a group of nodes that are probably related to one or several common origins is determined by the shifting relationships. We define shifting links for capturing the opportunity of moving from one to a latter event, which is used to measure the chances if a pair of nodes have a strong common origin (or several common origins). The shifting relationship in time and space is defined as a spatial and temporal weighting function. Spatial weights decrease with the increasing distance between the two nodes and the strength temporal weights raises after T1 until the middle of the range between T1 and T2 where the strength reaches a peak, and decreases after the middle point until T2. Therefore, the spatial weighting function is formulated as a distance-decay function with a threshold at D (Equation 2), and the temporal weighting equation is formulated as a bell shape function with the mean as the middle between T1 and T2 and the standard deviation as the half of the range between T1 and T2 (Equation 3). The results of the space-time weighting equations are illustrated in Fig. 4. The two weighting equations are normalized to range from zero (the lowest) to one (the highest). Thus, the shifting relationship between nodes is defined as a combined weight (${W}_{c}(i,j)$) of the occurrence of the link calculated based on the spatial and temporal weights with the Equations 2 and 3, respectively. We adopted the concept of Mantel Index, a commonly-used space-time correlation statistic which is based on a product of normalized spatial distances and time intervals^47^. Thus, the combined weight (Equation 1) adopts a product of spatial and temporal weights. A higher combined weight value between nodes indicates a higher space-time association with each other.

1 $${W}_{c}(i,j)={W}_{s}(i,j)\times {W}_{t}(i,j)$$

2 $${W}_{s}(i,j)=\{\begin{array}{cc}(1-\frac{d(i,j)}{D}{)}^{2} & \text{, if }d(i,j)\le D\\ 0 & \text{, otherwise}\end{array}$$

3 $${W}_{t}(i,j)=EXP(-\frac{{(t(i,j)-\frac{T1+T2}{2})}^{2}}{T2-T1})$$

where, ${W}_{s}(i,j)$, ${W}_{t}(i,j)$, and ${W}_{c}(i,j)$ are the spatial, temporal, and combined weighting functions; $d(i,j)$ and $t(i,j)$ are the spatial distance and temporal distance of the pair of nodes of a shifting link; $D$ is the spatial buffer; and *T*1 and *T*2 are the time-buffer and time-threshold.

For each target case(*j*), we compare the combined weight of each of its incoming shifting link(${W}_{c}(i,j)$) with the total combined weight of all of its incoming shifting links $(\sum _{k\in I(j)}{W}_{c}(k,j))$. The higher the relative weight of a shifting link, the more likely the target case is shifted from the shifting link.

4 $$RW(i,j)=\frac{{W}_{c}(i,j)}{{\sum }_{k\in I(j)}{W}_{c}(k,j)}$$

where, $RW(i,j)$ is the relative weight of shifting, $I(j)$ is the set of incoming shifting links of *j*.

Using the relative weight of shifting, we calculate the single propensity (${P}_{c}(s,(a,b))$) of each neighboring pair ($a,b$) from each common source ($s\in I(a)\cap I(b)$) with Equation 5. The total common propensity (${S}_{c}(a,b)$) of each neighboring pair from all of their common sources ($k\in I(a)\cap I(b)$) is calculated by Equation 6. Thus, the higher the total common propensity, the more likely the neighboring pair are shifted from one of their common sources.

5 $${P}_{c}(s,(a,b))=RW(s,a)\times RW(s,b)$$

6 $${S}_{c}(a,b)=\sum _{k\in I(a)\cap I(b)}{P}_{c}(k,(a,b))$$

where, ${P}_{c}(s,(a,b))$ is the single propensity from one common source to a pair of nodes of a neighboring pair; ${S}_{c}(a,b)$ is the total common propensity of a pair of neighboring nodes.

Based on the groups of total common propensity values, a non-parametric bootstrap procedure is used to identify pairs of neighboring relationships that are significantly stronger than the others. A critical value (${S}_{c}^{{\rm{^{\prime} }}}$) is calculated as the threshold, from a bootstrapping process, to filter the neighboring pairs. Denotes N as the number of neighboring pairs, the bootstrapping process randomly sample N-pairs of neighboring pairs ($a,b$), and calculates and records the mean (*mean*_*s*_) of the N-samples’ total common propensity (${S}_{c}(a,b)$). The resampling process is repeated for M times, and the bootstrapped mean (*mean*_*boot*_) and standard deviation ($s{d}_{boot}$) of the M-recorded means (*mean*_*s*_) is calculated for evaluating the critical value with Equation 7. Then, the neighboring pair with a common propensity $({S}_{c}(a,b))$ that is higher than or equal to the critical value(${S}_{c}^{{\rm{^{\prime} }}}$) is defined as the *cluster pair*. The other neighboring pairs would then be neglected in the following procedures.

7 $${S}_{c}^{{\rm{^{\prime} }}}=mea{n}_{boot}+1.28\times s{d}_{boot}$$

where, ${S}_{c}^{{\rm{^{\prime} }}}$ is the upper critical value from the bootstrapping process; $mea{n}_{boot}$ and $s{d}_{boot}$ are the mean and standard deviation that represents the distribution of the M-times of resample means of the total common propensity values (*mean*_*s*_). To calculate the upper bound of the 80% interval of the distribution (two-tails), the standard deviation is multiplied by 1.28 and summed to the mean of the M samples.

After all of the neighboring pairs are evaluated, we can construct a network where the nodes are events, and two nodes are connected if a cluster pair exists between them. We search for the connected components within the network, which represent the subgroup of nodes, in which each node has a connecting path to any node within the same subgroup. Each connected component is identified as a space-time sub-cluster. Therefore, a sub-cluster is composed of a group of events which are connected by a bunch of cluster pairs, indicating that they are more likely to have one or several common sources.

### Constructing the progression chains

The progressions represent the connections between sub-clusters, that show how the sub-clusters influence one another, and the direction of the diffusion process. Two scales of progressions are included in this part: the progression links, and the progression chains. After all cluster pairs are found, the most probable common origin (the source(*s*) of the shifting links with the max *P*_*c*_(*s*,(*a,b*))) of each cluster pair are revealed and defined as common links. *A progression link* is constructed between two sub-clusters that has at least one common link exists between them, which represents the progressions from one sub-cluster to another. And, the sub-clusters that are connected with each other form a *progression-chain* (Fig. 1b), whereas the other sub-clusters that are not linked with any sub-clusters are called *isolated sub-clusters*.

## Application: the sub-clusters and progressions of Dengue Fever in Taiwan from 1998 to 2015

To test and demonstrate our TaPiTaS algorithm for understanding and visualizing the diffusion process, we used Dengue Fever data in Kaohsiung City, Taiwan from 1998 to 2015. Located in East Asia, Taiwan straddles tropical and subtropical zones. The tropical weather pattern of Kaohsiung City, hot temperature and high humidity in summer, provides suitable habitats for the vector of Dengue Fever (mainly the *Aedes aegypti* mosquitoes)^57,58^. Moreover, Kaohsiung City, which has the largest harbor and an international airport in Taiwan, is a major East-Asian transport hub that has a high volume of travelers from South-East Asia. This increases the opportunity of Dengue Fever importation to Taiwan^59,60^. The number of Dengue Fever cases in Kaohsiung City shows an annual cyclical pattern, which mainly starts in the early summer and ends in the late winter of the next year^57^. Therefore, we have separated the data in this study to start from April 1st of each year to March 31st of the following year. The locations of past outbreaks are mostly concentrated in southern Taiwan (including Kaohsiung City) due to the temperature suitability for the mosquitoes breeding^61,62^. Therefore, the aim of the case study is to detect sub-clusters from the annual Dengue Fever cases and identify processes between the sub-clusters.

**Figure 5:**
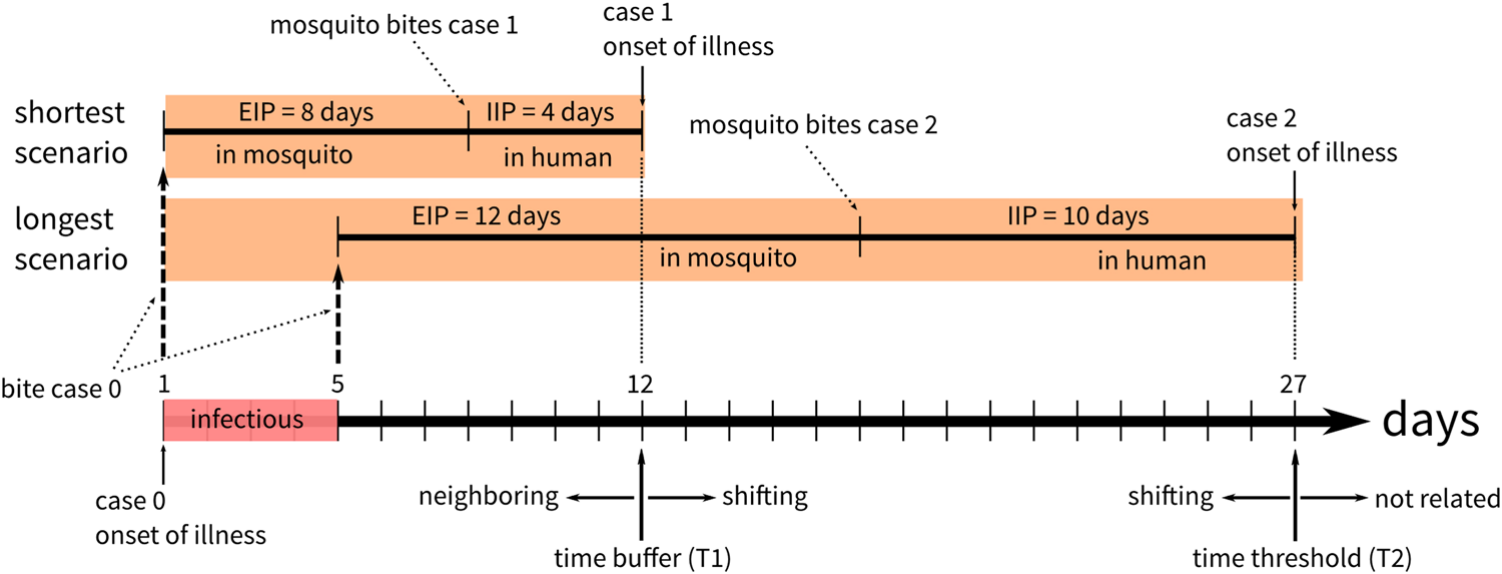
The time parameters settings were designed to capture the ranges of infectious period, extrinsic incubation period (EIP), and intrinsic incubation period (IIP) of Dengue Fever. The time buffer ranges from the first day when the case 0 is sick to the minimum days for EIP and IIP. The time threshold ranges beyond time buffer to the maximum days for EIP and IIP.

Dengue Fever is a vector-borne disease, with a human-mosquito-human transmission cycle. There is a temporal lag between the time a case is infected and the time when the case become infectious. The infectious period after the first symptoms appear is about 4–5 days; the extrinsic incubation period (EIP) for mosquitoes is about 8–12 days; and the intrinsic incubation period (IIP) for humans is about 4–10 days^63^ (Fig. 5). Therefore, the minimum temporal lag between a pair of related cases is 12 days (the first patient infects the mosquitoes on the first day, 8 days of extrinsic incubation period, following by 4 days of intrinsic incubation in the second patient), whereas the maximum time-lag is about 27 days (the first patient infects the mosquitoes on the 5th day, following by 12 days and 10 days of the extrinsic and intrinsic incubation period, respectively).

Our data was provided by Taiwan Centers for Disease Control (Taiwan CDC), which records the daily number of Dengue Fever cases in each basic statistical unit (BSU) in Kaohsiung City, separated into imported (to Taiwan) and local indigenous cases, based on the epidemiological investigation records. The Kaohsiung City area includes the previously named Kaohsiung City and Kaohsiung County until 2010. Only the indigenous case data was used in this study to eliminate the external importation noise from the local diffusion process. On average for the whole of Taiwan, each BSU contains 400 people. There are three main parameters in the our TaPiTaS algorithm: a spatial distance parameter, and two temporal parameters. In this case study, the spatial neighboring parameter (*D*) was set to 500 meters following Hsu and Tsai^64^. Based on the intrinsic and extrinsic incubation periods of Dengue Fever^63^, the time-buffer(*T*1) was set to 12 days; and the time-threshold parameter (*T*2) was set to 27 days. Experimentally, the bootstrapping value converged after 99 iterations.

### Descriptive statistics of events, pairs of close events, sub-clusters, and chains

Table 1 shows the descriptive statistics of the diffusion progression by year. The total number of cases varied between years: six out of the 18 years have less than 100 cases; in 2014 and 2015, the number of cases exceeded 10,000. The number of sub-clusters are related to the number of cases in the year, but are not always proportional to the number of cases. For example, 2010 had less cases than 2011, but more sub-clusters were detected. The sub-cluster size (SC size) and duration (SC duration) shows the overall extent and the duration of continuity of sub-clusters. Sub-cluster size is measured by the number of cases in every sub-cluster, whereas the duration is measured by the days between the first case and the last case in each sub-cluster. Regardless of the significant differences in the total number of cases, the median number and the median absolute deviation of the sub-cluster sizes and durations over the 18 years are similar: sub-clusters consist of about 2 to 4 cases, and the duration median is 5 days (with 4 days MAD). MAD is the abbreviation for median absolute deviation^65^, which better describes our range than the normal standard deviation.

**Table 1:** The descriptive statistics of the diffusion progression from 1998 to 2015, including cases, sub-clusters(SC), progression links(PL), and chains. The SC size represents the median number of cases within a sub-cluster; chain size represents the median number of sub-clusters within a chain; the SC duration represents the median temporal duration of the sub-clusters, which measures the number of days between the earliest and the latest cases within a sub-cluster; the chain duration represents the median temporal duration of the chains. The numbers in brackets are the median absolute deviation (MAD) of the corresponding columns.

year	no. cases	no. SC	SC size	SC duration	no. iso-SC	no. PL	no. chains	chain size	chain duration
1998	113	5	3 (0)	5 (4)	2	2	1	3.0 (0)	66.0 (0)
1999	20	1	—	—	1	0	0	—	—
2000	—	—	—	—	—	—	—	—	—
2001	220	9	8 (9)	14 (15)	3	8	1	6 (0)	81 (0)
2002	4671	276	3 (1)	7 (6)	52	198	39	2 (0)	36 (22)
2003	34	2	2 (0)	2 (2)	2	0	0	—	—
2004	56	1	—	—	1	0	0	—	—
2005	96	6	4 (1)	6 (4)	4	1	1	2 (0)	19 (0)
2006	955	102	3 (1)	4 (4)	21	57	25	3 (1)	29 (18)
2007	168	7	4 (1)	12 (9)	3	2	2	2 (0)	31 (1)
2008	417	31	3 (1)	5 (6)	18	7	6	2 (0)	23 (7)
2009	751	75	3 (1)	6 (6)	11	52	17	3 (1)	36 (16)
2010	1044	129	3 (1)	5 (4)	36	68	28	3 (1)	35 (16)
2011	1158	119	3 (1)	5 (6)	34	61	25	2 (0)	34 (16)
2012	478	37	3 (1)	5 (4)	18	15	6	2 (0)	26 (9)
2013	64	3	3 (0)	3 (1)	1	1	1	2 (0)	17.0 (0)
2014	15011	433	3 (1)	5 (4)	99	292	56	2 (0)	39 (19)
2015	19520	484	3 (1)	5 (4)	115	356	44	3 (1)	50 (36)

The number of isolated sub-clusters (iso-SC), progression links (PL), chains, and the characteristics of progression chains including the sizes and duration are also shown in Table 1. The chain size is measured by using the number of sub-clusters in each chain to show the extent of chains in the year; the chain duration is measured by using the temporal difference between the first case and the last case in each chain to show the temporal continuity in each year. Similar to the sub-cluster sizes and durations, the chain sizes and durations are also similar through the 18 years regardless of the differences in terms of the total size of cases. The chain sizes consist of 2 to 3 sub-clusters and the duration median is one month (with 2 weeks MAD).

### Visualizing the processes of diffusion

From the descriptive statistics analysis (Table 1), in three years, 2002, 2014, and 2015, Kaohsiung City experienced the severest epidemics in the past 70 years^65,66^. The total number of confirmed Dengue Fever cases in these three years were 4671 cases, 15011 cases, and 19520 cases, respectively. The progression structures of large-scale Dengue transmission in these epidemic years can be distinctly illustrated and investigated quantitatively for demonstrating the functionality of our proposed algorithm. Therefore, in the following discussion, we focused on the three years. In 2002, our algorithm found 276 sub-clusters and 224 of these formed 39 progression chains. In 2014, 435 sub-clusters were detected, and 336 of these sub-clusters were found in 56 progression chains. In 2015, 484 sub-clusters were detected, and 368 formed 44 progression chains.

**Figure 6:**
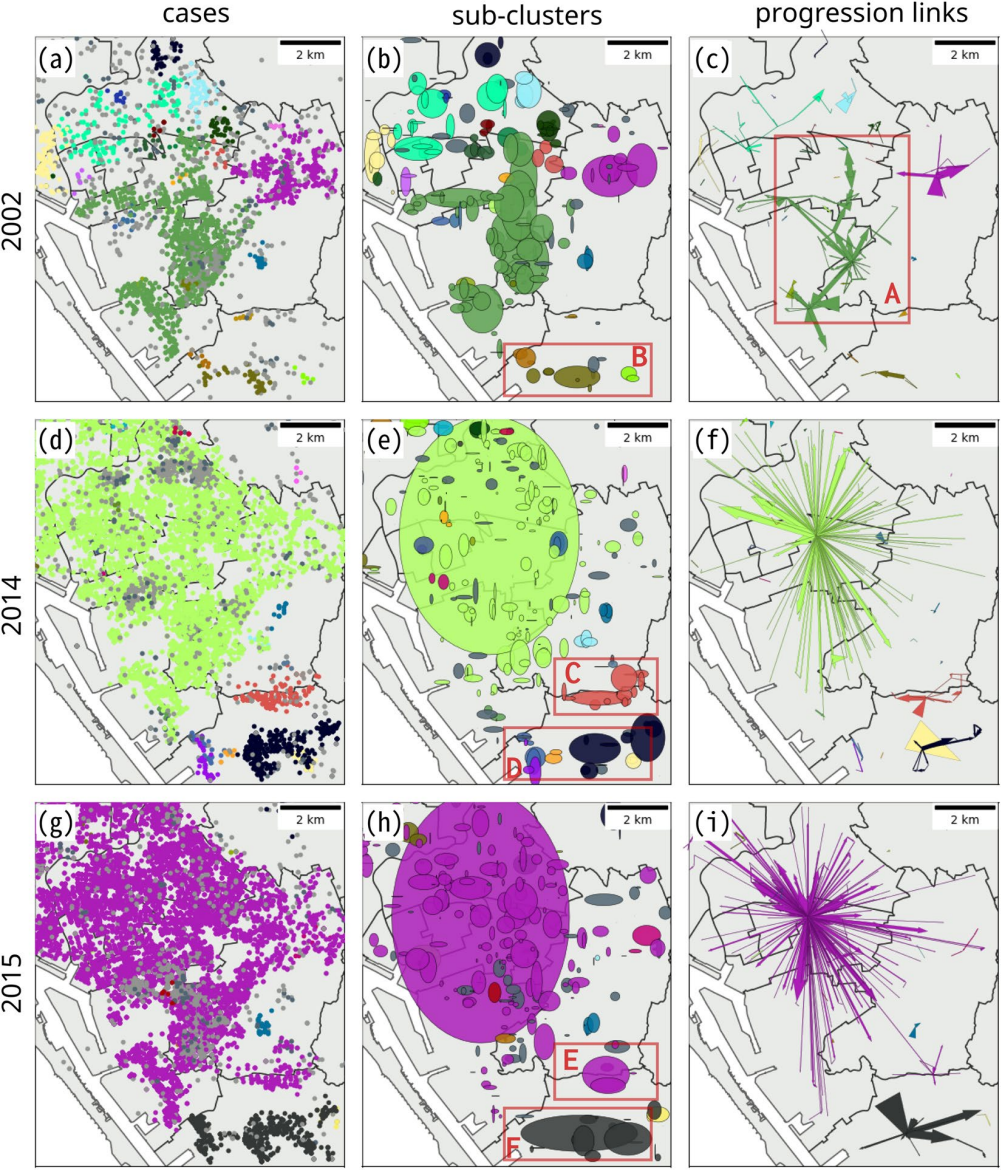
Spatial distribution of cases (left), sub-clusters (middle), and progression links (right) in 2002 (first row), 2014 (second row), and 2015 (third row). The colors differentiate the progression chains. The sub-clusters were determined﻿ by the standard ellipses’ standard distances (long and short axes) and the orientation. The number of shifting links that form the progression links were used to determine the width of the progression links. The maps were created using Matplotlib (2.0.0) package in Anaconda (4.0.0, Python 2.7.11 version, url: https://www.continuum.io/anaconda-overview).

The spatial distribution of the cases, sub-clusters and progression links in 2002, 2014, and 2015 are shown in Fig. 6. The colors indicate the progression chains to which they belong. The sub-clusters are presented as standard ellipses using the XY coordinates of cases to determine the standard distances. The width of the progression links indicates the number of shifting links that connect the two sub-clusters. Cases were distributed throughout the city for the three years (Fig. 6a,d,g). In 2002, the algorithm found a significant spatial separation between the progression chains (Fig. 6a). Figure 6b shows that the progression chains were differentiated into ellipses by our algorithm based on the temporal differences between the cases. In order to show the evolution and strength between the sub-clusters, the progression links were mapped in Fig. 6c.

The spatial distribution of the progression chains in 2014 and 2015 were different from the year 2002, in that an extremely large sub-cluster appeared and covered the most highly populated area of the city (Fig. 6e,h). This is because the cases were not concentrated in a small area over the small temporal duration, but distributed over a larger region within the spatial search radius. Different from 2002, some smaller progression chains overlapped with the large sub-cluster in 2014. However, most of the smaller overlapping sub-clusters were linked with another large sub-cluster in 2015. Comparing the 2014 results to the 2015 results, the 2015 analysis revealed more sub-clusters but less progression chains. The chain sizes in 2015 were larger than those in 2014, indicating that the sub-clusters in 2015 were more connected to one another. For example, in the southern part of Kaohsiung City (lower part of the map), five progression chains were detected in 2014, but only one progression chain was found in 2015.

## Discussion

When exploring diffusion processes, especially for disease diffusion, the actual interactions between events (cases) are normally not known, since information about the transmission process (sub-cluster evolution over time) between large numbers of cases is difficult to measure retrospectively. The location and temporal case information is normally the only available data. To uncover any diffusion process from this type of data, we have developed the TaPiTaS algorithm for exploring and visualizing space-time point data to show the process of diffusion. TaPiTaS is a novel algorithm that utilizes the temporal lag within the diffusion process and the spatial distance between events to detect the spatial-temporal sub-clusters and to uncover the development of progression chains. Recall that a sub-cluster represents events that have high propensity to have common origins in the diffusion process. A progression chain represents the linked sub-clusters. Thus TaPiTaS is an important conceptual contribution to cluster structure and sub-cluster evolution understanding.

We anticipate that this algorithm can be used to explore diffusion phenomena, in which temporal lag is a key to the movement of the diffusion process. Unlike previous methods for detecting clusters that only included a time-window to capture the temporal proximity, our algorithm adds a lagged time-window (between T1 and T2) to capture the shifts between events, and weights the temporal lags to search for the sub-clusters and progressions. This enables us to distinguish the sub-clusters from the spatially and temporally clustered events.

To demonstrate the analysis process and algorithm outputs, we presented a case study, of the annual Dengue Fever diffusion process in Kaohsiung City, Taiwan, from 1998 to 2015. In the case study, despite varying sizes of epidemics in different years, we detected similar size sub-clusters with a median of 2 to 4 cases. The reason that most of the sub-clusters are composed of small numbers of cases is because the algorithm distinguishes the relationships between space-time proximate events using the lagged time-window, and the algorithm filtered neighboring pairs using a critical value to ensure that only the pairs of neighbors with compelling high total common propensity are used to detect sub-clusters. In other words, by connecting the events that happen in the same place at the same time (with buffer zones in space and time), our TaPiTaS algorithm aggregates cases into small groups, namely the sub-clusters, which could form part of a larger space-time cluster. Moving on to discuss the progression chains, by connecting the sub-clusters according to the evolution of shifting links, TaPiTaS uncovers the movement progression in space-time dimensions, namely the processes within a space-time cluster.

Our results differentiate diffusion structures in time and space among the severest epidemic years: 2002, 2014, and 2015. It implies Dengue Fever epidemics for these years in Kaohsiung could have been triggered by different sources of infection, driving forces of transmission, and the effectiveness of intervention measures. In 2002, its diffusion structure indicated the epidemic circulated around the different district-level administration areas. The district heath authority is the basic operation unit for disease control and prevention. Thus, it implies that the peripheral areas may lack consistent intervention measures. The sub-clusters with green color originated from the box-A (Fig. 6c), near to the boundary of the Fengshan district, a frequent Dengue-epidemic region^57,67^, then spreads south-west, north-west, and north. Fengshan district is a satellite city of the Kaohsiung metropolitan area that has a high population density but its socio-economic level is relatively lower than the neighboring central business district (CBD) of Kaohsiung City. Recent studies have shown the areas with high urbanization levels, high population density, and low social-economic status would increase the risk of Dengue diffusion and tend to become the sources of diffusion^52^. Moreover, there is a group of small and less connected sub-clusters appearing in the southern part (Fig. 6b, box-B) of the map, indicating that the areas may also be vulnerable to Dengue Fever and may require more attention in the future.

The epidemic progressions in 2014 and 2015 share some common diffusion structure characteristics. First, these two Dengue epidemics are composed of two major progression chains. One of the progression chains contains the largest size of sub-cluster, and these cases in the sub-cluster were mostly located in epidemic areas (Fig. 6e,h). Second, two smaller groups of progression chains appear in the southern part of Kaohsiung City, one of the progression chain groups contains the orange progression chain in 2014 (Fig. 6e, box-C), and part of the purple progression chain in 2015 (Fig. 6h, box-E). The other contains a group of progression chains in 2014 (Fig. 6e, box-D), and a black progression chain in 2015 (Fig. 6h, box-F). These locations are approximately the same vulnerable areas as identified in 2002 (Fig. 6b, box-B). Therefore, comparing these progression chains could reveal geographic epidemiological links among these three years. On the other hand, we also identified different progression directions in 2014 and 2015. The progression links in 2014 pointed northwest are stronger than the other directions; however, in 2015, some of the progression links pointed south are stronger. In 2014, the epidemic sub-clusters mainly diffused northward and north-westward, whereas in 2015, some significant southward spreading of sub-clusters was also observed.

Using standard ellipses to represent sub-clusters, and arrows between them to show the progression chains, the diffusion process is illustrated. Previous studies on space-time clustering issues using the space-time scan statistic (SaTScan) visualized the clustered area with a circle, and described the clustering periods of each cluster^68,69^. This visualization method shows a clear location and the magnitude of the detected clusters. But it ignores the process of cluster development. Other space-time clustering studies used kernel density techniques to visualize the clustered area in space-time cube plots superimposed on a 2D X,Y map^21,22^. Event temporal mobility was illustrated on the Z or 3rd dimension, and thus the event migration could be shown on a three-dimensional figure. However, without the ability to interact with a static 3D image, it is confusing to understand cluster progression. Furthermore, using density based techniques, simple temporal proximity relationships were assumed, not calculated from the data whereas in our TaPiTaS algorithm, the temporal lag between cases is explicitly included in the calculation procedure. In our study, by using standard ellipses to visualize sub-clusters, we suggest that readers can get a better understanding about the shape and directions of sub-clusters; using arrows to link the sub-clusters and to represent the progression links with the day of appearance of the sub-clusters, the process of diffusion can be revealed.

Profiling diffusion structures of Dengue Fever epidemics can provide important clues for health authorities to implement spatially-targeted intervention measures. Our proposed algorithm reveals three key geospatial characteristics of Dengue diffusion by identifying progression links: the source areas, the target areas, and the linkage between epidemic areas. The source areas of diffusion may represent the areas that are suitable for the breeding of disease vector. Eliminating the habitats of mosquitoes could be beneficial for the source area and also its adjacent areas. It would produce the trans-boundary externalities in disease control^70,71^. The target areas could be the regions with spatial risk factors of Dengue transmission. The areas with these risk factors are expected to be more vulnerable to the Dengue Fever transmission, including high urbanization levels, low social-economic status, favorable weather conditions and previous epidemic records^52,72,73^. The linkage between epidemic areas indicates the spatial interactions or communication through human movement. It also implies the possible route of the virus diffusing from one to another region. A longer progression linkage may cause a large-scale epidemic through long-distance human moving behaviors^50,66,74^.

Our TaPiTaS algorithm has several limitations. First, when a large amount of events happens within a short spatial distance of each other, over a short temporal period, such as occurred in the case study in 2014 and 2015 with more than 15,000 cases per year, our algorithm could not fully capture the diffusion process. Instead of identifying sub-clusters as separate events, our algorithm grouped them as a giant sub-cluster and which became a common source for the sub-clusters at different places. We think that this situation requires further investigation to understand the mechanism of the extreme sub-clusters or progression chains. Second, no significance evaluation procedure is included in our TaPiTaS algorithm. While clusters could happen by chance under a complete spatial randomness condition, the detected sub-clusters and the progression links could be a result of a space-time random distribution. To overcome this limitation, a Monte-Carlo significance test could be included to evaluate the level of significance of each sub-cluster and progression link. Third, the underlying population distribution is not considered. Other methods, such as SaTScan, consider the distribution of population at risk to capture the spatial inhomogeneity of population, and perform a risk normalization procedure in the calculation. Risk normalization is not included within our current algorithm, but it may be done in the data preparation stage before the algorithm, or in the results evaluation stage using additional calculation after the algorithm. Fourth, the definition of spatial proximity is measured only by the straight line distance between events, which simplifies the concept of distance. The measurement of distance is a key issue in most spatial analysis models and methods. Some alternatives, such as street network distance, time needed for movement, or cost of movement, could be used alternatively to measure the spatial proximity between events. Fifth, we used two equations to calculate the spatial and temporal weight separately (Equations 2 and 3), and multiplied them to calculate the integrated weight (Equation 1) in the algorithm. The equations may not be suitable for all events of interest in different studies. Therefore, the parameters for weighting spatial and temporal distance, and for the combined weight of shifting links could be modified according to the needs of the study subject.

## Conclusion

Temporal lag is essential in the understanding of diffusion processes. We have proposed a novel algorithm, TaPiTaS, that utilizes the spatial distance and temporal interval between events, to explore and to visualize the diffusion process. This algorithm can be used to explore point data with timestamps, and outputs sub-clusters, progression links, and progression chains to show the process of diffusion. As a method for space-time point data exploration, TaPiTaS differentiates the relationships of events and detects the sub-clusters of events that are immediately proximate to each other. TaPiTaS then identifies the directional links between sub-clusters, which represents diffusion progressions. By additionally visualizing the detected sub-clusters and progression links, our TaPiTaS algorithm contributes a more detailed and in-depth understanding of the geographic diffusion process then currently exists. In summary, we propose our TaPiTaS algorithm as a tool for uncovering the evolution of space-time clusters of entities.

### Data availability

The data that support the findings of this study are available from Center for Disease Control, Taiwan, under the Taiwan Open Government Data License, version 1.0 (http://data.gov.tw/license#eng). The datasets are available in the Daily reported Dengue Fever cases since 1998 repository (dataset url: http://data.gov.tw/node/21025, direct download url: http://data.gov.tw/iisi/logaccess/61136?dataUrl=http://nidss.cdc.gov.tw/download/Dengue_ Daily_EN.csv&ndctype=CSV&ndcnid=21025).

## Electronic supplementary material

The spatial and temporal distributions of Dengue Fever in 2002.

The spatial and temporal distributions of Dengue Fever in 2014.

The spatial and temporal distributions of Dengue Fever in 2015.
